# Identification of Novel *Staphylococcus aureus* Core and Accessory Virulence Patterns in Chronic Rhinosinusitis

**DOI:** 10.3390/ijms26083711

**Published:** 2025-04-14

**Authors:** Simon P. Goldie, Laurie C. Lau, Huw A. S. Jones, Philip G. Harries, Andrew F. Walls, Rami J. Salib

**Affiliations:** 1 School of Clinical and Experimental Sciences, Faculty of Medicine, University of Southampton, Southampton SO16 6YD, UK; simongoldie.sg@googlemail.com (S.P.G.); l.c.lau@soton.ac.uk (L.C.L.); a.f.walls@soton.ac.uk (A.F.W.); 2 Department of Otorhinolaryngology/Head & Neck Surgery, University Hospital Southampton NHS Foundation Trust, Southampton SO16 6YD, UK

**Keywords:** *Staphylococcus aureus*, chronic rhinosinusitis, biofilms, virulence factors, quorum sensing

## Abstract

*Staphylococcus aureus* (*S. aureus*) colonizes the nasal cavities of both healthy individuals and patients with chronic rhinosinusitis (CRS) with (CRSwNP) and without (CRSsNP) nasal polyps. Treatment-resistant *S. aureus* biofilms and intracellular persistence are common in CRS patients, requiring the expression of specific virulence factor genes to transition into these forms. We hypothesized that *S. aureus* isolates from non-diseased controls, CRSsNP patients, and CRSwNP patients would exhibit distinct virulence factor patterns contributing to persistence and intracellular survival in CRS patients. Nasal swabs from seventy-seven individuals yielded *S. aureus* cultures in eight non-diseased controls, eight CRSsNP patients, and five CRSwNP patients. Whole-genome sequencing analyzed stress, antimicrobial resistance, and virulence genes, including plasmids and prophages. Four virulence factor gene patterns emerged: a core set (*hlgA*, *icaC*, *hlgB*, *hlgC*, *hld*, and *aur*) present in all isolates, and accessory sets, including the enterotoxin gene cluster (*seo*, *sem*, *seu*, *sei*, and *sen*) and a partial/complete invasive virulence factor set (*splE*, *splA*, *splB*, *lukE*, and *lukD*) (*p* = 0.001). CRSwNP isolates exhibited incomplete carriage of the core set, with frequent loss of *scn*, *icaC*, and *hlgA* (*p* < 0.05). These findings suggest that *S. aureus* has clusters of virulence factors that may act in concert to support the survival and persistence of the bacteria, resulting in enhanced pathogenicity. This may manifest clinically with resistant disease and refractoriness to antibiotics.

## 1. Introduction

*Staphylococcus aureus* (*S. aureus*), a Gram-positive commensal bacterium, can be associated with a wide spectrum of pathology, ranging from asymptomatic colonization of the nares to being the leading cause of nosocomial bacteremia with an associated mortality of 15–60% [1]. Due to the diverse phenotypic behavior of *S. aureus*, it has been difficult to characterize its involvement in diseases such as chronic rhinosinusitis (CRS). *S. aureus* colonizes the nasal cavity in 64% of patients with nasal polyps (CRSwNP) compared with 33% of those without polyps (CRSsNP) and 20% of those without CRS [2,3]. A higher proportion of patients with CRSwNP demonstrate IgE towards *S. aureus* enterotoxins in their serum than those without CRS (22.6–32.5% vs. 6.7–14.3% of controls) [4]. Culture of *S. aureus* pre- and post-operatively in patients with CRS is a poor prognostic indicator for disease recurrence and recalcitrance [5]. However, the factors responsible for the enhanced pathogenicity of *S. aureus* strains prevalent in difficult-to-treat CRS disease remain poorly understood.

*S. aureus* can persist in the nasal cavity of both asymptomatic carriers and CRS patients, evading host immune responses and the effects of antimicrobial therapies [6]. In CRS, *S. aureus* appears to frequently adopt invasive strategies, including localizing intracellularly within host cells and forming extracellular biofilms, both of which are facilitated by the expression of specific virulence factors [7,8,9,10]. These virulence factors can be broadly categorized into three main groups: adherence factors, pore-forming toxins, and superantigens [11]. Adherence factors, or adhesins, enable *S. aureus* to bind to components of the host extracellular matrix. Key adhesins include staphylococcal protein A, fibronectin binding proteins A and B, and clumping factors A and B. These proteins play crucial roles in the initial attachment to host tissues and production of biofilms [12,13]. Pore-forming toxins include α toxin, leukocidins, β-hemolysin, and phenol-soluble modulins. These toxins disrupt host cell membranes and host cell components, leading to cell lysis and immune evasion. α toxin exhibits broad lytic activity against epithelial and endothelial cells, T cells, monocytes, macrophages, and platelets, as well as contributes to phagosome escape [14]. Leukocidins lyse neutrophils and erythrocytes, while β-hemolysin has cytotoxic effects on keratinocytes, polymorphonuclear leukocytes, monocytes, and T lymphocytes and is also implicated in biofilm development and phagosomal escape [11,15,16]. Phenol-soluble modulins are cytotoxic to both red and white blood cells and contribute to biofilm structuring and detachment [17,18]. Superantigens are potent T-cell mitogens that bind the variable β-chain of the T-cell receptor, triggering widespread T-cell activation, B-cell proliferation, increased local IgE production, and a robust type 2 inflammatory cytokine response. Staphylococcus enterotoxin B, a well characterized superantigen, has been shown to recruit mast cells to the epithelial and subepithelial layers of CRS patients’ nasal explant tissues and promote mast cell *S. aureus* uptake and degranulation [11,19,20].

Before the widespread adoption of whole-genome sequencing, *S. aureus* virulence and strain outbreaks were often inferred using accessory gene regulator (*agr*) locus typing and staphylococcal protein A gene (*spa*) typing, both of which provide insights into the virulence potential [21,22,23]. The *agr* locus plays a key role in regulating the expression of numerous *S. aureus* virulence factors. Loss-of-function or frameshift mutations within this region have been associated with the emergence of small colony variants (SCVs), which exhibit reduced virulence due to diminished expression of these factors [24]. Recent evidence has shown an increased number of mobile genetic elements (MGEs), including prophages and plasmids, in CRS isolates, providing additional virulence and antimicrobial resistance genes that may well support *S. aureus* survival and enhance pathogenicity [25,26].

Given the different selection pressures and distinct environments between the non-diseased, CRSsNP, and CRSwNP nasal mucosa, we hypothesized that differences in stress, antimicrobial resistance, and virulence genes exist among *S. aureus* isolates cultured from each condition, contributing to enhanced pathogenicity of the bacteria and manifesting clinically as disease recalcitrance and treatment resistance. To test this, we conducted a prospective investigation of *S. aureus* isolates cultured from control, CRSsNP, and CRSwNP patients, analyzing the genomic profile and the associated MGEs for stress, virulence, and antimicrobial resistance genes.

## 2. Results

### 2.1. Patient Demographics

Nasal swabs were obtained from 77 patients enrolled in the study, including 30 control subjects, 20 patients with CRSsNP, and 27 patients with CRSwNP. *S. aureus* was cultured in eight control subjects, eight CRSsNP patients, and five CRSwNP patients. A significantly higher proportion of asthmatic individuals was observed within the CRSwNP group (55%) compared with the control (13.3%) and CRSsNP (20%) groups (*p* = 0.001). Additionally, oral steroid use within the month preceding sample collection was significantly higher in the CRSwNP group (22.2%) compared to controls (6.67%) and CRSsNP patients (0%) (*p* = 0.03). Smoking prevalence was greater in the control group (13.3%), with no current smokers identified in either the CRSsNP or CRSwNP groups (*p* = 0.04). Other demographic variables did not differ significantly between the groups (Table 1).

A subgroup analysis was performed on subjects from whom *S. aureus* was cultured. A significantly higher Modified Lund–Mackay Score (MLMS) was observed for patients with *S. aureus* colonization (mean score 12.0) compared to those without *S. aureus* (mean score 7.5, *p* = 0.04). No other statistically significant differences were identified (Table 2).

### 2.2. Bacterial Genome Sequencing

Illumina short-read, paired-end sequencing was performed on twenty-one *S. aureus* isolates, including eight from control subjects, eight from CRSsNP patients, and five from CRSwNP patients. Paired-end reads were assembled and analyzed using the Bactopia bacterial alignment and analysis pipeline [27]. The assembled genome sizes spanned between 2.67 and 3.61 Mbp.

### 2.3. Staphylococcal Protein A and agr Loci and Frameshift Assessment

An analysis of the highly variable region of *spa* identified three duplicated *spa* types: t008, t015, and t571. Two of these were shared between the control and CRSwNP groups, while the third was found in both the control and CRSsNP groups.

The *agr* locus was predominantly type I and found in 67% of the isolates. Type II was observed in 19% and type III in 14% of isolates (*n* = 21). One isolate from the CRSwNP group displayed a frameshift insertion mutation in the *hld* gene (encoding the delta hemolysin toxin) while another showed an absent *hld* gene (Table 3).

### 2.4. Stress, Antimicrobial Resistance, and Virulence Genes

**Figure 1:**
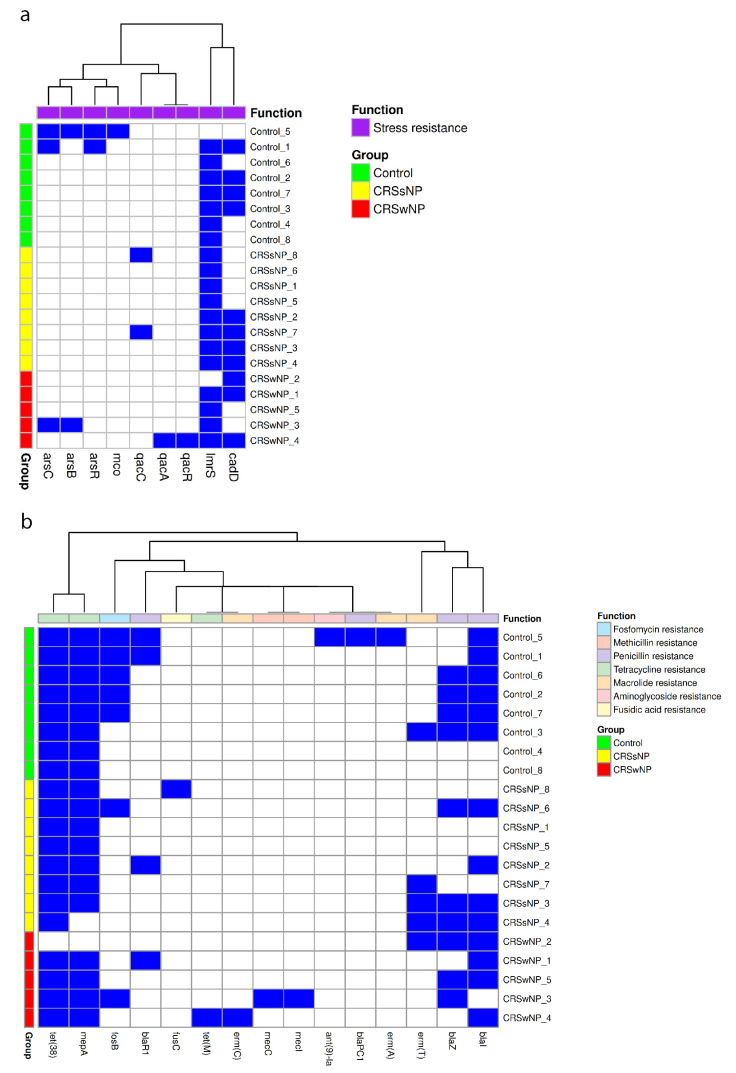
Stress and antimicrobial resistance gene carriage in control, CRSsNP, and CRSwNP *S. aureus* isolates. Each heatmap demonstrates the presence (blue) or absence (white) of (**a**) stress and (**b**) antimicrobial resistance genes from each isolate.

Minimal differences were observed between the patient groups when comparing stress genes. The *lmrS* gene was present in 90% of isolates, while *cadD* was identified in 52%, with no statistically significant differences in genes detected using a Chi-square test (Figure 1a). Antimicrobial resistance genes exhibited greater variability, with *tet(38)* found in 95% of isolates and *mepA* in 90%, both conferring tetracycline resistance (*n* = 21). Penicillin resistance genes (*blaI*, *blaZ*, *blaPC1*, and *blaR1*) were identified in 75% of control isolates (*n* = 8), 50% of CRSsNP isolates (*n* = 8), and 100% of CRSwNP isolates (*n* = 5). The macrolide, lincosamide, and streptogramin resistance genes *erm(T)* and *erm(A)* were identified in 25% of control isolates (*n* = 8), 37% of CRSsNP isolates (*n* = 8), and 20% of CRSwNP isolates (*n* = 5). The presence of the fosfomycin resistance gene *fosB* approached significance (*p* = 0.08), with 62.5% of control isolates harboring this gene (*n* = 8) compared to 12.5% of CRSsNP isolates (*n* = 8) and 20% of CRSwNP isolates (*n* = 5) (Figure 1b).

Four dominant virulence gene clusters were identified. All isolates contained the majority of a core set of virulence genes, including *hlgA*, *icaC*, *hlgB*, *hlgC*, *hld*, and *aur*. This core set was identified alone or supplemented by accessory virulence factor genes such as the enterotoxin gene cluster (*seo*, *sem*, *seu*, *sei, and sen*) or a full or partial cluster of invasive virulence genes (*splE*, *splA*, *splB*, *lukE,* and *lukD*). Among the isolates from the control group, 50% contained the invasive virulence genes and 37.5% carried the enterotoxin gene cluster (*n* = 8). Within the CRSsNP group, 25% of isolates showed a partial presence of the invasive virulence gene cluster, while 12.5% demonstrated an enterotoxin gene cluster (*n* = 8). In the CRSwNP group, 60% showed a complete enterotoxin gene cluster and 40% showed the invasive gene cluster (*n* = 5). The *hld* gene was absent in the CRSwNP2 isolate and had previously exhibited a frameshift mutation in CRSwNP3.

**Figure 2:**
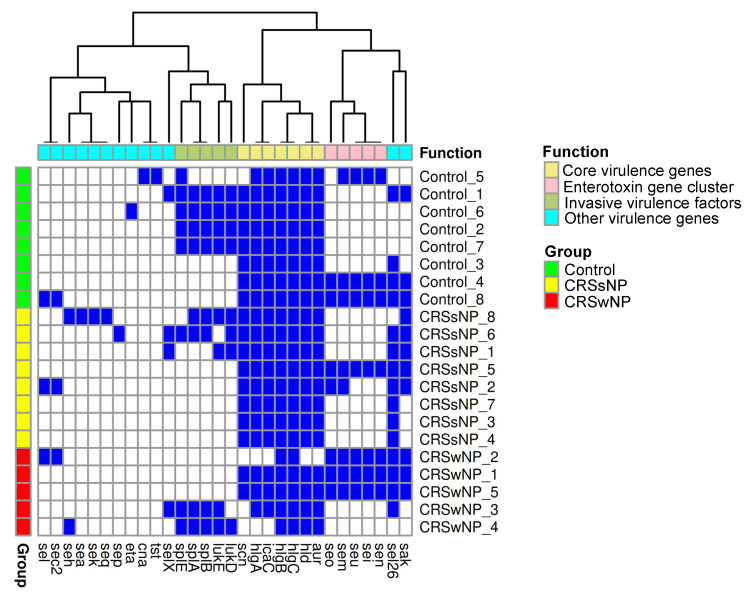
Virulence gene carriage in control, CRSsNP, and CRSwNP *S. aureus* isolates. The heatmap demonstrates the presence (blue) or absence (white) of virulence genes in each isolate.

The Chi-square analysis revealed a statistically significant absence of *hlgA* in 40% of CRSwNP isolates (*p* = 0.03), *icaC* was absent in 40% of CRSwNP isolates (*p* = 0.03), and *scn* was absent in 60% of CRSwNP isolates and one control isolate (*p* = 0.02) (Figure 2).

**Figure 3:**
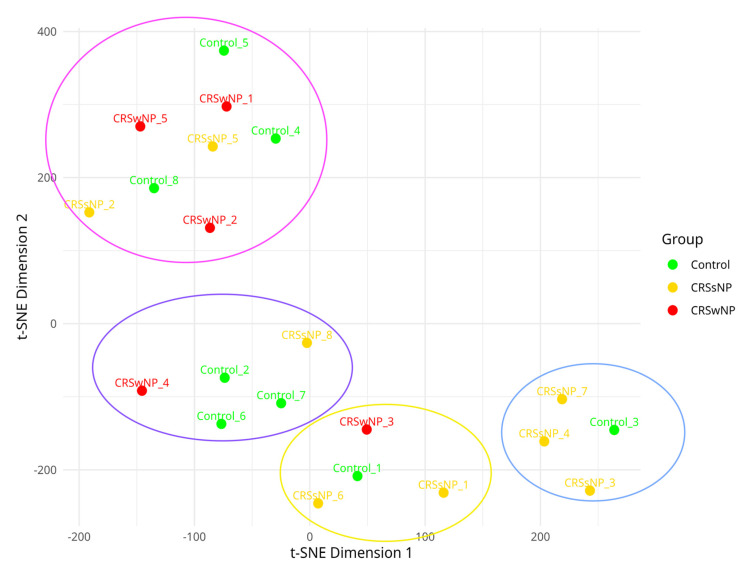
T-distributed stochastic neighbor embedding of virulence factor presence and absence matrix demonstrating the clustering of virulence factor patterns. Isolates are labelled using their titles. The blue bubble represents the core virulence factors alone, and the pink bubble represents the core and enterotoxin gene cluster. The yellow bubble represents a partial invasive virulence gene cluster, and the purple bubble represents the core and invasive virulence gene clusters.

The cluster analysis method t-SNE confirmed the presence of the four distinct virulence gene cluster patterns (Figure 3). An initial PERMANOVA test for the significance of clusters based on the control, CRSsNP, and CRSwNP groups showed no significant associations (*p* = 0.26). However, when testing the significance of our four distinct virulence gene cluster patterns, PERMANOVA revealed a significant result (*p* = 0.001).

### 2.5. Mobile Genetic Element Identification

**Figure 4:**
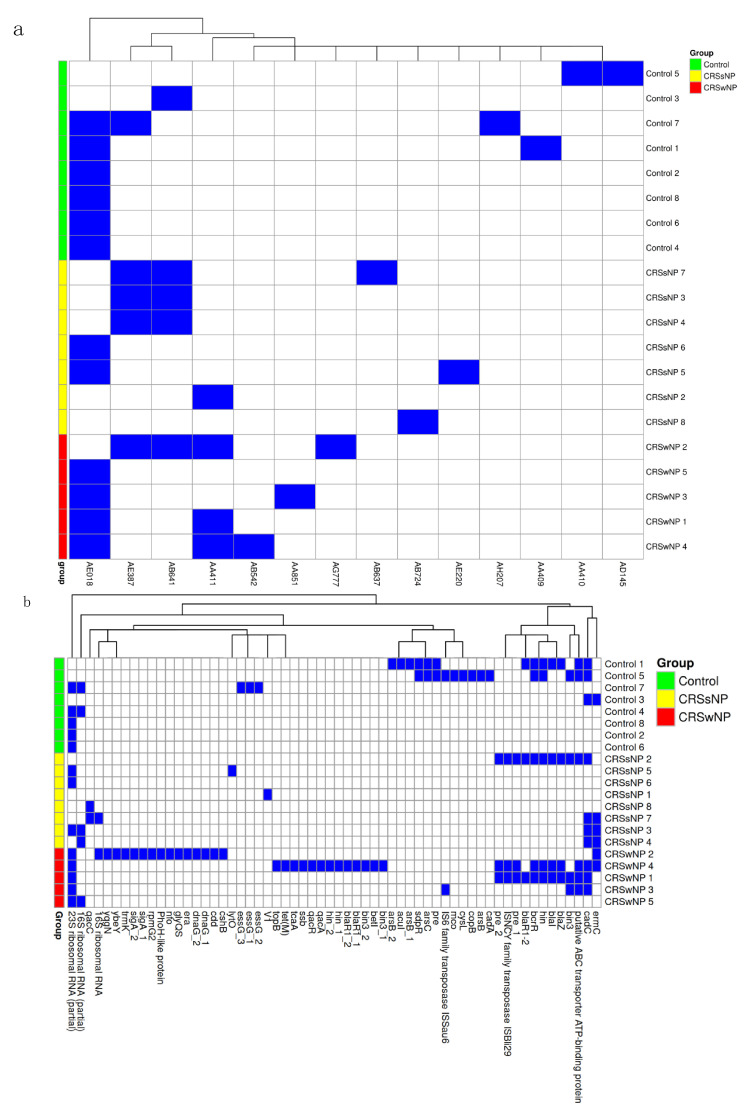
Plasmid identification and genes. (**a**) Heatmap of Blast high-scoring pairs from MOB-suite. The presence of HSP is indicated by the blue rectangle and its absence is indicated by the white rectangle. (**b**) Heatmap of sequenced genes from each identified plasmid listed by group (blue = present and white = absent).

The mean number of plasmids in each group was calculated, demonstrating an average of 1.4 plasmids per isolate in the control group, 1.7 in the CRSsNP group, and 2.4 in the CRSwNP group; however, this difference was not statistically significant using one-way ANOVA (*p* = 0.15). Fourteen plasmid clusters were identified based on Blast high scoring pairs (HSPs), with no statistically significant differences between the plasmid cluster distribution and disease group (Figure 4a).

We further examined the individual genes encoded by plasmids. On average, plasmids contributed 15.87 genes to control isolates, 10.53 to CRSsNP isolates, and 30.6 to CRSwNP isolates, with no significant differences between groups (one-way ANOVA, *p* = 0.27). Approximately 59–67% of the identified genes were either hypothetical or not characterized. Notably, several antimicrobial resistance genes were detected on plasmids, including *ermC*, *blaZ*, *blaI*, *blaR1*, and *tet(M)*. Erythromycin resistance genes were more frequently located on plasmids than in the core genome, appearing in one control, three CRSsNP, and two CRSwNP isolates. The penicillin resistance gene *blaZ* was found on plasmids from one control, one CRSsNP, and two CRSwNP isolates, none of which exhibited these genes in the core genome. Plasmids appeared to contribute few stress and virulence genes, with no clear concentration in any group (Figure 4b).

**Figure 5:**
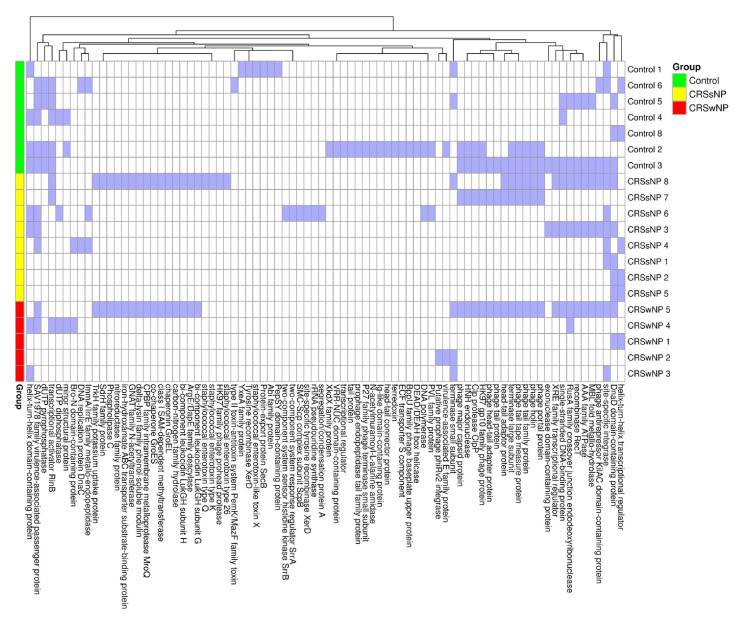
Heatmap of PhiSpy with the identified phage genes listed by group (blue = present and white = absent).

The mean number of prophages in each group was calculated, demonstrating 2.38 prophages per CRSsNP isolate, compared to 1.86 in control isolates and 1.60 in CRSwNP isolates; this difference was not significant using one-way ANOVA (*p* = 0.35). We also assessed the number of prophage genes per isolate, finding an average of 44.0 prophage genes in control isolates, 34.5 in the CRSsNP isolates, and 34.4 in the CRSwNP isolates. A large proportion of identified genes were hypothetical or not characterized (70–68%). Subsequently, we mapped known prophage genes to assess their contributions to virulence. The resulting heatmap showed a sparse distribution with no significant differences between the groups (Figure 5).

## 3. Discussion

Our findings provide a detailed assessment of the *S. aureus* genome and MGEs in isolates from the middle meatuses of CRS patients with comparisons to disease-free control subjects and may have important implications for our understanding of virulence and antimicrobial resistance.

The demographic characteristics of our cohort align with those of previous studies, showing a significantly higher prevalence of asthma in the CRSwNP group compared with the CRSsNP and control groups [28,29]. Inhalant allergy prevalence was also elevated in the CRSwNP group (29.6%) compared to the CRSsNP (15.0%) and control (26.6%) groups, with higher rates across all groups relative to other studies, likely due to recruitment from surgical lists and rhinology clinics [28]. The MLMS was significantly higher in the CRSwNP group (13.5) compared to the CRSsNP (7.9) and control (3.6) groups, as expected based on the European Position Paper on Rhinosinusitis and Nasal Polyps 2020 diagnostic criteria, and were similar to previously reported values [30,31]. Smoking incidence was significantly higher in our control group, possibly due to primary prevention counselling in the CRS and asthma groups [29,32].

*S. aureus* colonization in the CRSwNP group was lower than expected, at 18.5%, although some studies have observed similarly low rates [3,33]. This discrepancy may be attributed to higher antimicrobial use in the CRSwNP group (23.5%). The subgroup analysis revealed a significant association between *S. aureus* colonization and a higher MLMS, consistent with other reports [34].

The *S. aureus agr* locus regulates the production of RNAII, RNAIII, and downstream virulence factors, functioning as a quorum-sensing system. Numerous studies classify the *agr* locus into groups I-IV based on polymorphisms in the *agrB*, *agrC,* and *agrD* genes, which influence virulence factor expression and are related to specific disease manifestations [21]. Most isolates belonged to group I, which is typically associated with commensal carriage, urinary infections, and bacteremia, with no significant differences detected between the groups [35,36]. The *hld* gene forms part of the RNAIII transcript and *agr* operon, and its absence or frameshift mutation abolishes the transcription of the accessory gene regulator [*agrA*] and downstream virulence factors [37]. The *hld* gene was absent or showed a frameshift mutation in 40% of CRSwNP isolates but was present in all other groups. Mutations in the *agr* locus and *hld* gene have been shown to create senescent bacteria known as small colony variants (SCVs), which show superior intracellular translocation in epithelial cells [38,39,40]. Furthermore, *hld* mutations have been shown to create larger biofilms in comparison to those with a functional *hld* gene [41]. Stress gene patterns across all groups displayed no significant differences. The penicillin resistance gene *blaZ* was present in 75% of control isolates, 25% of CRSsNP isolates, and 100% of CRSwNP isolates when reviewing the core and plasmid genomes. These findings contrast with those of Jervis Bardy et al., as they detected a persistent rate of *blaZ* carriage of 57% in both the CRSsNP and CRSwNP groups, suggesting differences in selection pressures between the studies [42]. The clustering analysis suggested the presence of four recurring virulence factor patterns, potentially reflecting distinct modes of pathogenicity across the different groups. These included a core set of virulence factors alone, a core set with an enterotoxin gene cluster, or a core set with either a partial or complete invasive virulence factor cluster. Notably, no isolates contained both the enterotoxin and invasive virulence factor clusters simultaneously.

The core set, including *hlgA, hlgB, hlgC, icaC*, *scn*, *hld* and *aur*, was present in nearly all isolates, though some CRSwNP isolates exhibited a partial loss of the core genes. The bi-component gamma-hemolysins (*hlgB* and *hlgC*) were well conserved across all groups. However, the *hlgA* gene was significantly absent in 40% of CRSwNP isolates [43]. The immune modulator aureolysin (*aur*), which cleaves the C3 complement protein, was present in all groups but absent in a single CRSwNP isolate [44]. The intercellular adhesion gene *icaC*, part of the intracellular adhesion (*ica)* locus responsible for biofilm formation, was unexpectedly absent in 40% of CRSwNP isolates, though it was present in the other groups. The *icaC* gene product mediates the transfer of polysaccharide intercellular adhesion molecules to the cell membrane, facilitating the development of longer poly-N-acetylglucosamine oligomers and larger biofilms [45,46]. The staphylococcal complement inhibitor, typically found on the immune evasion cluster (*scn*) of MGEs, was absent in 60% of CRSwNP isolates. The *scn* gene product binds and inactivates the complement component C3b, which is a potent activator of B cells. Consequently, *scn* gene absence could contribute to the increased IgE response against *S. aureus* seen in CRSwNP patients’ nasal tissues [3,25].

In addition to core virulence factors, an accessory cluster of invasive virulence factors was identified, consisting of *lukE*, *lukD*, *splE*, *splA*, and *splB*. This gene cluster was fully present in 50% of control isolates and 40% of CRSwNP isolates, and partially present (i.e., missing one or more genes) in 37.5% of CRSsNP isolates. The *lukE/D* gene product leukocidin E/D induces calcium channel activation in neutrophils, leading to cell death and compromised local immunity [43,47]. The *splA* gene product cleaves the mucin 16 glycoprotein, which forms a defensive mucosal barrier, while the *splB* gene product inhibits complement activation by cleaving several complement components (C3–C9), blocking opsonophagocytosis and the terminal complement cascade [48]. The target of *splE* remains unclear.

The enterotoxin gene cluster of superantigens (*sei*, *sem*, *sen*, *seo*, and *seu*) formed a further accessory group of virulence factors. They were present in 37.5% of control isolates, 60% of CRSwNP isolates, and 12.5% of CRSsNP isolates, with a further CRSsNP isolate showing partial presence. These enterotoxins are associated with long-term colonization in the nasal airway, cystic fibrosis lungs, and atopic dermatitis wounds [49]. They stimulate T-cell proliferation but appear to have unexpectedly low levels of immunoglobulins raised towards them in human serum when compared with other superantigens [49]. The precise function of these enterotoxins is yet to be fully elucidated.

Plasmid carriage sequentially increased from control to CRSsNP and CRSwNP isolates. Others have demonstrated an increasing plasmid copy number over time in *S. aureus* cultured from CRS patients, increasing their ability to produce antimicrobial-resistant biofilms [26]. We therefore studied the mobile genes each plasmid provided, identifying tetracycline, macrolide, and penicillin resistance genes. There appeared to be no differences in known plasmid-encoded genes between groups; however, a significant number of genes were classified as hypothetical or of unknown function. As a result, the functional significance of any observed differences might be difficult to interpret due to the presence of these genes with uncharacterized functions to date. We identified a lower carriage of prophages in the CRSwNP group than in the control and CRSsNP groups. Prophage encoded genes relevant to *S. aureus* virulence were sparsely detected, but there were no antimicrobial resistance genes identified on prophages, in keeping with others’ results [25]. The detection of these genes was infrequent and did not significantly contribute to the overall pattern of virulence factors previously noted.

This study has some limitations. Patient demographic data, including medication history, were recorded by questionnaire. Therefore, a one-month timeframe was used to record antibiotic and steroid use in order to reduce recall bias. It is possible that longer-term exposures could have influenced *S. aureus* carriage, antimicrobial resistance and virulence factor patterns, which warrant further study. While our findings highlight potential differences in antimicrobial resistance and virulence gene patterns between patients with CRS phenotypes and controls, the small sample size limits the strength of conclusions that can be drawn. This limitation was partly a result of our sampling technique, which prioritized the recovery of viable *S. aureus* isolates for future downstream experimental analyses, including transcriptomic and proteomic validation. Sequencing large numbers of genes often reveals significant heterogeneity, complicating statistical significance testing. More subtle differences may have been overlooked, which might have been identified in larger cohorts. Reflecting on our results, taking multiple swabs of each patient’s middle meatus may have identified multiple *S. aureus* isolates with different virulence factor patterns that could work synergistically. Furthermore, culture of the sinonasal tissue may have boosted the *S. aureus* culture yield; however, it would have been unethical to harvest tissue from control participants due to the inherent risks associated with the procedure. Nevertheless, our findings complement existing sequencing data and contribute to a more detailed understanding of mechanisms that enhance *S. aureus* pathogenicity in CRS patients.

## 4. Materials and Methods

### 4.1. Subjects

Patients undergoing a nasal endoscopic examination in rhinology clinics and those undergoing endoscopic sinus surgery procedures by the senior authors (H.A.S.J., P.G.H., and R.J.S.) at University Hospital Southampton between 22 April 2021 and 9 August 2022 were invited to participate. Subjects that met the European Position Paper on Rhinosinusitis and Nasal Polyps 2020 [30] criteria for the diagnosis of CRS were stratified into CRSsNP and CRSwNP, and those with other diagnoses, including allergic rhinitis, nasal masses, and anatomical nasal obstruction, were placed in the control group. The exclusion criteria included patients under 18 years of age; those with cystic fibrosis, primary ciliary dyskinesia, or immune deficiency syndromes; those with an inability to provide informed consent; and those with blood-borne viruses, including hepatitis B, hepatitis C, and human immunodeficiency virus. Demographic data, including age, sex, atopic status, antibiotic and steroid use in the past month, medical history, history of asthma, and smoking habits, were collected by questionnaire. Nasal swabs were taken either intraoperatively or under topical anesthetic from the middle meatal region in the clinic. Ethical approval was obtained for the study via the National Health Service (UK), London—Hampstead Research Ethics Committee (REC: 20/PR/0183). All participants provided written informed consent.

### 4.2. Specimen Testing and Genomic Sequencing

Bacterial swabs (M40 transystem, Copan, Brescia, Italy) were spread onto *S. aureus* 24 h brilliance agar plates (Oxoid, Basingstoke, UK). Blue coagulase-positive colonies were tested for catalase and DNase and subjected to MALDI-TOF to confirm their identity as *S. aureus* species. Positive *S. aureus* cultures were grown to logarithmic growth in Rosewell Park Memorial Institute Medium 1640 (Life Technologies, Paisley, UK) at 37 °C and frozen in the presence of 25% glycerol (VWR, Lutterworth, UK) at −80 °C. All 21 collected *S. aureus* strains were prepared in RNA Shield (Zymo Research, Freiburg im Breisgau, Germany) and underwent 30× Illumina short-read sequencing (MicrobesNG, Birmingham, UK).

### 4.3. Alignment and Assembly of the Genome

Twenty-one paired-end sequencing reads were assembled and annotated using Bactopia (v3.0.0). Quality control was performed using Bbtools (v38.96), Fastp (v0.23.2), FastQC (v0.11.9), and Lighter (v1.1.2), followed by genome assembly with Shovill (v1.1.0) and annotation with Prokka (v1.14.6) and Bakta (v1.8.2) [50,51,52,53].

### 4.4. Typing of the spa and agr Loci and Detection of Frameshifts

The Bactopia workflow staphtyper was used to analyze our assemblies [54]. This elucidated the staphylococcal protein A gene (*spa*) type and accessory gene regulator (*agr*) locus type. The *agr* locus was further investigated by aligning our sequences to the *S. aureus* Newman strain (GenBank accession: GCA_040702955.1) using snippy (v4.6.0), followed by manual inspection using Integrated Genome Viewer (v2.18.2) [55].

### 4.5. Stress, Virulence, and Antimicrobial Resistance Genes

Stress, virulence, and antimicrobial resistance genes were assessed using the Bactopia workflow AMRFinderPlus (v3.11.18) [56]. Detected genes were imported into the statistical package R (v4.3.1) [57]. Genes with greater than 90% sequence coverage and homology were transformed into presence/absence matrices. Isolates were then clustered within their respective groups using complete linkage clustering and heatmaps were generated using the pheatmap package (v1.0.12) [58].

The patterns of virulence factor genes were further analyzed using t-distributed stochastic neighbor embedding (t-SNE) in R using the Rtsne package (v0.17) and subsequently visualized using ggplot2 (v3.4.0) [59,60]. The significance of clusters was assessed by PERMANOVA using the vegan package (v2.6-10) [61].

### 4.6. Plasmid Detection

Plasmids were detected using the Bactopia workflow MOB-suite (v3.1.7), generating a Blast HSP table for each isolate along with a FASTA file of each plasmid’s sequence [62,63]. The FASTA files were interrogated using Prokka (v1.14.6) to generate a list of the genes present in each plasmid [52]. The resulting data had hypothetical genes removed and were transformed into a gene presence/absence matrix. This matrix was subsequently visualized using pheatmap [58].

### 4.7. Prophage Detection

Prophages were detected using PhiSpy (v4.2.21) executed with the –-output_choice 9 flag, providing the number of prophages and predicted phage-associated genes from the genome [64]. The resulting predicted prophage genes were used to create a presence/absence matrix, which was visualized using the pheatmap package [58].

### 4.8. Statistical Analysis

A statistical analysis of patient demographics was performed using SPSS (v29.0.2.0, IBM, Portsmouth, UK). Data normality was assessed through histogram plots and normality tests. Pearson’s Chi-square test and one-way ANOVA were used to compare categorical and numerical demographic data, respectively. Differences in genomic information were analyzed in R, with categorical data such as gene presence assessed using the Chi-square analysis and numerical data assessed using one-way ANOVA [57].

## 5. Conclusions

In summary, our findings support the presence of a core virulence factor set consisting of *hlgA*, *hlgB*, *hlgC*, *hld*, *icaC*, *aur*, and *scn*, which is detected in nearly all *S. aureus* isolates, but appears to be incompletely carried in some CRSwNP isolates, with the loss of *scn*, *icaC*, and *hlgA*. The loss of these genes may support bacterial survival by facilitating the formation of SCVs and biofilms and the promotion of IgE production through B-cell activation. This core virulence set is observed both independently and in conjunction with accessory virulence factor sets, including the enterotoxin or complete/partial invasive virulence gene clusters. These previously unrecognized virulence patterns likely represent distinct phenotypes that act synergistically in promoting bacterial colonization and persistence, and manifest clinically with disease recalcitrance and refractoriness to antibiotics.

## Figures and Tables

**Table 1:** Demographic profile of the study population.

Disease Type	Control	CRSsNP	CRSwNP	*p*-Value
Number of subjects	30 (38.9%)	20 (25.9%)	27 (35.1%)	-
Gender (M/F)	15:15	9:11	16:11	0.603 ^†^
Mean age (SD)	53.5 (16.6)	57.2 (16.7)	51.4 (15.9)	0.447 ^‡^
Clinical review/procedure	Clinical review 6 Biopsy 4 Septoplasty/PETS 13 Limited FESS 7 Full house FESS 0	Clinical review 7 Biopsy 0 Septoplasty/PETS 1 Limited FESS 6 Full house FESS 6	Clinical review 5 Biopsy 0 Septoplasty/PETS 0 Limited FESS 2 Full house FESS 20	<0.001 ^†^
Antibiotic use in the past month	2 (6.67%)	1 (5.0%)	5 (18.5%)	0.225 ^†^
Steroid use in the past month	2 (6.67%)	0	6 (22.2%)	0.033 ^†^
*S. aureus* culture	8 (26.6%)	8 (40%)	5 (18.5%)	0.262 ^†^
Airborne allergies	8 (26.6%)	3 (15%)	8 (29.6%)	0.490 ^†^
Asthma	4 (13.3%)	4 (20%)	15 (55.5%)	0.001 ^†^
Ex-smokers	5 (16.7%)	5 (25%)	7 (25.9%)	0.628 ^†^
Current Smokers	4 (13.3%)	0	0	0.039 ^†^
Modified Lund–Mackay Score	3.6	7.9	13.5	<0.001 ^‡^

Study population demographics (^†^ Pearson’s Chi-square test for categorical data, ^‡^ one-way ANOVA for numerical data). The *p*-value where a Pearson Chi-square test was used, quantifies the relationship of the categorical variable (e.g., antibiotic use) between the control, CRSsNP and CRSwNP groups. The *p*-value of one-way ANOVA quantifies the relationship of numerical data (e.g., age) between the control, CRSsNP and CRSwNP groups. PETSs (Powered Endoscopic Turbinoplasties) and FESS (Functional Endoscopic Sinus Surgery).

**Table 2:** Demographic profile based on a sub-analysis of the *S. aureus* culture status.

Culture of *S. aureus*	No Growth	Growth	*p*-Value
Number of subjects	56 (73%)	21 (27%)	-
Gender (M/F)	31:25	9:12	0.328 ^†^
Age (SD)	49.3 (15.6)	51.0 (18.5)	0.981 ^‡^
Clinical review/procedure	Clinical review 11 Biopsy 2 Septoplasty/PETS 9 Limited FESS 14 Full House FESS 20	Clinical review 7 Biopsy 2 Septoplasty/PETS 5 Limited FESS 0 Full House FESS 7	0.090 ^†^
Antibiotic use in the past month	7 (12.5%)	1 (4.8%)	0.322 ^†^
Steroid use in the past month	7 (12.5%)	1 (4.8%)	0.322 ^†^
Airborne allergies	14 (25%)	5 (23.8%)	0.914 ^†^
Asthma	14 (25%)	9 (42.9%)	0.127 ^†^
Ex-smokers	11 (19.6%)	6 (28.5%)	0.423 ^†^
Current smokers	3 (5.4%)	1 (4.8%)	0.904 ^†^
Modified Lund Mackay Score	7.5	12.0	0.038 ^‡^

Subgroup analysis of patients with a positive *S. aureus* culture and their demographics (^†^ Pearson’s Chi-square test, ^‡^ one-way ANOVA). The *p*-value where a Pearson Chi-square test was used, quantifies the relationship of the categorical variable (e.g., antibiotic use) between the control, CRSsNP and CRSwNP groups. The *p*-value of one-way ANOVA quantifies the relationship of numerical data (e.g., age) between the control, CRSsNP and CRSwNP groups. PETSs (Powered Endoscopic Turbinoplasties) and FESS (Functional Endoscopic Sinus Surgery).

**Table 3:** *spa* and *agr* types and assessment of the *agr* operon.

Isolate	*agr* Locus Type	*spa* Type	Frameshifts Mutations in the *agr* Operon
Control 1	I	t008	None
Control 2	II	t084	None
Control 3	I	t1451	None
Control 4	I	t065	None
Control 5	III	t338	None
Control 6	II	t346	None
Control 7	II	t084	None
Control 8	I	t230	None
CRSsNP 1	I	t1236	None
CRSsNP 2	I	t340	None
CRSsNP 3	I	t571	None
CRSsNP 4	I	t571	None
CRSsNP 5	I	t065	None
CRSsNP 6	I	t008	None
CRSsNP 7	I	u	None
CRSsNP 8	III	T127	None
CRSwNP 1	I	T015	None
CRSwNP 2	I	T015	Absent *hld* gene
CRSwNP 3	II	T6292	Frameshift insertion at position 28 in the *hld* gene
CRSwNP 4	III	T127	None
CRSwNP 5	I	T18906	None

*S. aureus* isolates cultured from the control (green), CRSsNP (yellow), and CRSwNP (red) groups of patients. The accessory gene regulator (*agr*) locus type and staphylococcal protein A (*spa*) types for each group are displayed. An assessment of the *agr* operon is displayed. u = unrecognized by the program SPAtyper.

## Data Availability

Paired-end reads of the reported *S. aureus* strains and supporting data have been deposited in the European Nucleotide Archive at EMBL-EBI under accession number PRJEB81780 “https://www.ebi.ac.uk/ena/browser/view/PRJEB81780 (accessed on 28 October 2024)”.

## Abbreviations

The following abbreviations are used in this manuscript:

*agr*	Accessory gene regulator
ANOVA	Analysis of Variance
*aur*	Aureolysin gene
*blaZ*	Beta-lactamase gene
*cadD*	Cadmium resistance gene
CRS	Chronic rhinosinusitis
CRSsNP	Chronic rhinosinusitis without nasal polyps
CRSwNP	Chronic rhinosinusitis with nasal polyps
DNA	Deoxyribonucleic acid
*erm(A)*, *erm (T)*, *erm (C)*	Erythromycin resistance methylase genes
*fosB*	Fosfomycin resistance gene
*hlgA*, *hlgB*, *hlgC*	Gamma hemolysin genes
HSP	High scoring pairs
*hld*	Delta-hemolysin gene
*icaC*	Biofilm-associated intercellular adhesion gene
*lmrS*	Lincomycin resistance protein of *S. aureus*
*lukE*, *lukD*	Leukocidin genes
MALDI-TOF	Matrix-assisted laser desorption/ionization time of flight
MGE	Mobile genetic element
MLMS	Modified Lund–Mackay Score
PERMANOVA	Permutational multivariate analysis of variance
RNA	Ribonucleic acid
RNAII	Second transcript of the *agr* operon
RNAIII	Third transcript of the *agr* operon
*S. aureus*	*Staphylococcus aureus*
SCV	Small colony variant
*sei*, *sem*, *sen*, *seo*, *seu*	Enterotoxin gene cluster
*spa*	Staphylococcal protein A gene
*splA*, *splB*, *splE*	Serine protease genes
*tet(38)*, *tet(M)*	Tetracycline resistance genes
t-SNE	t-distributed stochastic neighbor embedding
